# Microcracking of strawberry fruit cuticles: mechanism and factors

**DOI:** 10.1038/s41598-023-46366-8

**Published:** 2023-11-08

**Authors:** Grecia Hurtado, Moritz Knoche

**Affiliations:** Institute of Horticultural Production Systems, Leibniz University Hanover, Herrenhäuser Straße 2, 30419 Hannover, Germany

**Keywords:** Developmental biology, Physiology, Plant sciences

## Abstract

Microscopic cracks in the cuticle (microcracks) are the first symptom of the strawberry fruit disorder ‘water soaking’ in which the fruit surface appears watery, translucent, and pale. Water soaking severely impacts fruit quality. The objective was to investigate the factors and mechanisms of cuticular microcracking in strawberry. Fluorescence microscopy revealed numerous microcracks in the achene depressions, on the rims between depressions and at the bases of trichomes. Microcracks in the achene depressions and on the rims were either parallel or transversely oriented relative to a radius drawn from the rim to the point of attachment of the achene. In the achene depression, the frequency of microcracks with parallel orientation decreased from the calyx end of the fruit, towards the fruit tip, while the frequency of those with transverse orientation remained constant. Most microcracks occurred above the periclinal cell walls of the epidermal cells. The long axes of the epidermal cells were primarily parallel-oriented. Microcracking increased during fruit development. Cuticle mass per fruit remained constant as fruit surface area increased but cuticle thickness decreased. When fruit developed under high relative humidity (RH) conditions, the cuticle had more microcracks than under low RH conditions. Exposing the fruit surface to increasing RHs, increased microcracking, especially above 75% RH. Liquid-phase water on the fruit surface was markedly more effective in inducing microcracking than high vapor-phase water (high RH). The results demonstrate that a combination of surface area growth strain and water exposure is causal in inducing microcracking of the strawberry cuticle.

## Introduction

The cuticular membrane (CM) is an extracellular lipophilic membrane deposited on all primary above ground plant surfaces. The receptacle of the strawberry, a false fruit, is no exception. The CM comprises epicuticular and embedded cuticular waxes, polymeric cutin and phenolics. It is encrusted with cell-wall polysaccharides^1,2^. The CM fulfills important barrier functions. It helps restrict uncontrolled water and gas exchanges, inhibits pathogen entry and preserves fruit quality^3,4^. Maintaining all these functions requires an intact CM throughout fruit development.

The integrity of fruit cuticles is sometimes compromised by growth strains, resulting from rapid increases in fruit surface area and/or hostile environmental factors. Cuticular microcracks result from the failure of an overly-strained CM. Microcracks are minute fractures in the cuticle, not visible to the naked eye^5^. Microcracking is economically important in commercial fruit production and marketing. Microcracks impair the cuticle’s barrier function, thereby compromising fruit quality and, hence, fruit quantity (by necessary rejection). Moreover, microcracking is the first visible symptom in a number of fruit-surface disorders such as russeting in apple and pear^6^ and mango^7^, cracking in sweet cherry^8^ and grapes^9^, neck shrivel in plum^10^ and desiccation of lychee^11^. In strawberries, microcracking is the first symptom of impending water soaking^12^ and of cracking^13^. Microcracking allows rapid water uptake by viscous flow, that bypasses the cuticle barrier. The incidence of water soaking and cracking is high in open-field cultivation of strawberry and is substantially eliminated in protected cultivation^14^. Microcracking also increases fruit transpiration and, hence, accelerates fruit shrivel^12^. Lastly, microcracks serve as entry points for fruit rot pathogens causing diseases such as grey mold and anthracnose^15^. Despite the importance of cuticle integrity to the highly perishable strawberry fruit, remarkably little is known about cuticle deposition and microcrack formation in strawberries.

The objective of this study was: (1) to characterize microcracking of the cuticle of strawberry skin and (2) to identify the key factors affecting cuticular microcracking.

## Results

### Characterizing microcracking

**Figure 1:**
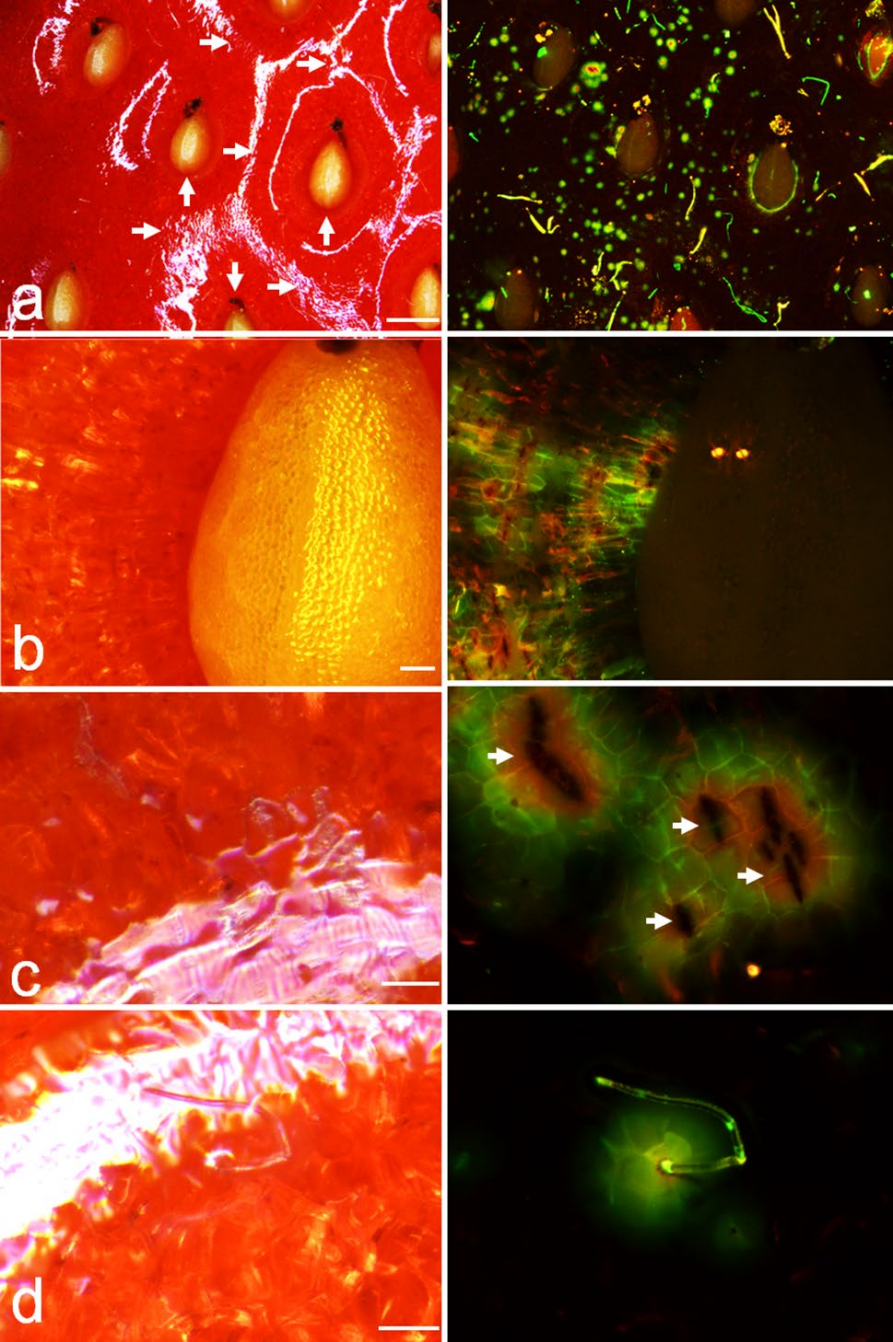
Micrographs of ripe ‘Clery’ strawberry fruit surface with numerous microcracks in the cuticle. Each row represents a pair of images in incident bright (left column) and fluorescent light (right column). (**a**) Overview of surface; (**b**) detail view of an achene depression; (**c**) detail view of a rim between achene depressions (**d**) detail view of trichomes. Scale bars in a = 1 mm and b,c,d = 0.1 mm. The vertical arrow in (**a**) indicates position of achene depression, the horizontal arrow in (**a**) the position on the rim between two adjacent achene depressions. Arrows in (**c**) point out microcracks. Fluorescence images were obtained following 5 min incubation of fruit in the fluorescent tracer acridine orange. The tracer does not penetrate an intact cuticle, but penetration is restricted to openings in the cuticle such as microcracks. Orange, yellow and green fluorescence indicates decreasing concentrations of fluorescent tracer. For details see materials and methods.

Fluorescence microscopy of the strawberry fruit (receptacle) surface revealed numerous microcracks (Fig. 1a). Microcracking occurred in the achene depressions and also on the rims between neighboring depressions (Fig. 1b,c); they also occurred at the base of trichomes as indicated by the orange, yellow or green fluorescence of acridine orange (Fig. 1d). The orange colors indicate high penetration underneath and in the immediate vicinity of a microcrack in the achene depression (Fig. 1b,c), whereas the green color is indicative for low penetration for example at the base of trichomes (Fig. 1d).

**Figure 2:**
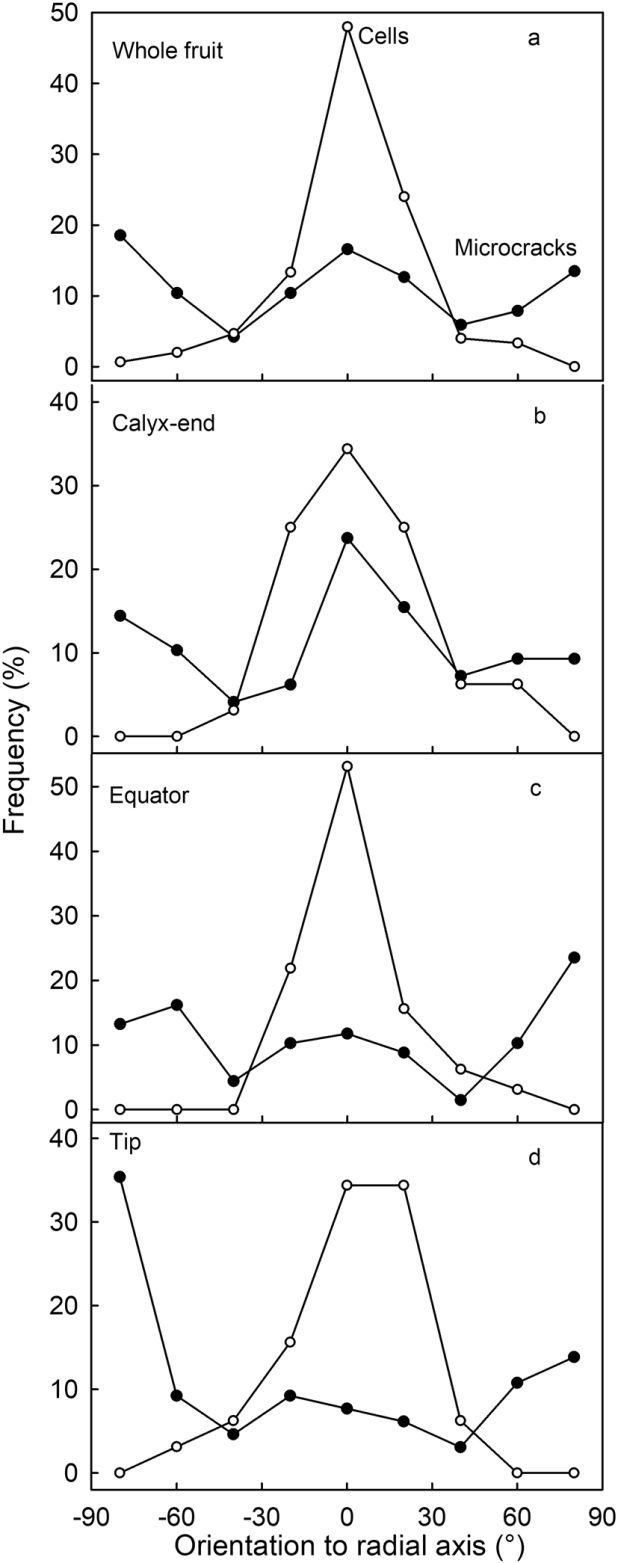
Frequency distribution of orientation of microcracks and epidermal cells in the achene depression of ‘Florentina’ strawberry. The orientation of microcracks and epidermal cells was measured relative to a radius drawn through the point of attachment of the achene. (**a**). Data were pooled for different regions. (**b**) Calyx region of the fruit (calyx and surroundings) within the seed zone; (**c**) Equator region (maximum fruit diameter and the center of the fruit). (**d**) Tip region of the fruit.

Irrespective of whether an achene was situated in the distal or proximal region of the fruit, microcrack orientation in all achene depressions showed a frequency distribution with three dominant peaks: at + 90°, 0° and −90° (Supplementary Fig. S1). This indicates most microcracks are oriented in one of two directions: either transverse to, or parallel to, a radius drawn between the depression rim and the point of attachment of the achene (Fig. 2a). Moving from the proximal (calyx) towards the distal (tip) end of the fruit, crack orientation changed systematically. The frequency of microcracks orientated parallel to the depression radius decreased, while that orientated transversely to it remained about constant (Fig. 2b–d). The frequency distribution of epidermal cell orientation in the achene depressions was simpler—there were no changes, proximal to distal. The long axes of all epidermal cells were oriented parallel to a radius drawn from the point of achene attachment to the rim (Fig. 2).

**Figure 3:**
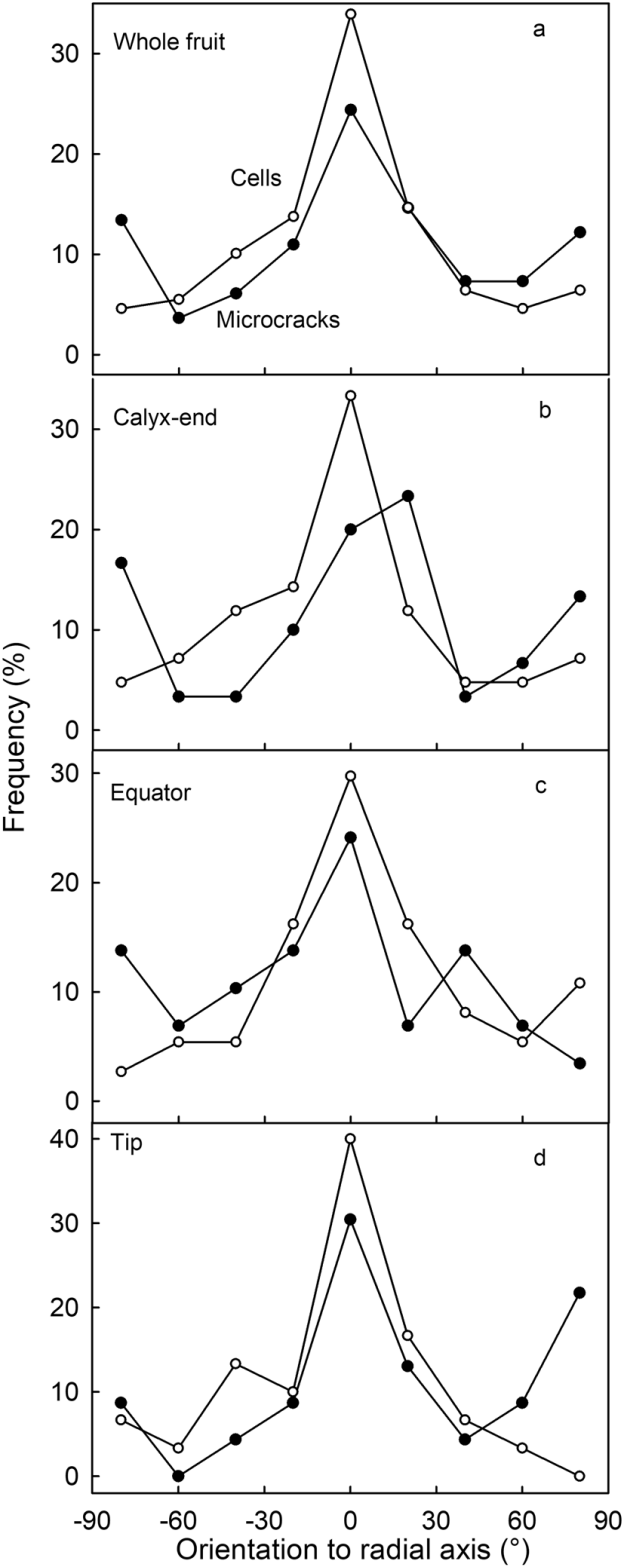
Frequency distributions of orientations of microcracks and epidermal cells on the rim between achene depressions of ‘Florentina’ strawberry. The orientation of microcracks and epidermal cells was measured relative to a radius drawn through the point of attachment of the achene. (**a**). Data pooled for different regions. (**b**) Calyx region of the fruit (calyx and surroundings) within the seed zone; (**c**) Equator region (maximum fruit diameter and the center of the fruit). (**d**) Tip region of the fruit.

On the rims between the depressions, microcrack orientations was similar to that in the achene depressions—microcracks were oriented either parallel to, or transverse to, a radius drawn to the point of attachment of the nearest achene. Location on the fruit surface had no effect. Meanwhile, the epidermal cell long-axis orientation was primarily parallel to a radius drawn to the point of attachment of the nearest achene, without major differences between regions of the fruit surface (Fig. 3). There were no systematic differences in the dimensions of the achene depressions between the proximal, equatorial or distal regions of the fruit surface (Supplementary Table S1).

There were no significant relationships between microcrack characteristics and fruit size but microcracking incidence did tend to decrease distally (*P* = 0.06; Supplementary Fig. S2). Microcracking was more frequent above the periclinal cell walls of the underlying epidermal cells, compared with above the anticlinal cell walls (Table 1).

**Table 1:** Frequency distribution of the location of cuticular microcracks above the anticlinal and periclinal cell walls in the achene depression and in the rim between achene depressions of strawberry ‘Florentina'.

Zone	Frequency distribution (%)	
	Periclinal	Anticlinal
Achene depression	60.2	39.8
Rim between achene depressions	65.7	34.3

### Factors affecting microcracking

**Figure 4:**
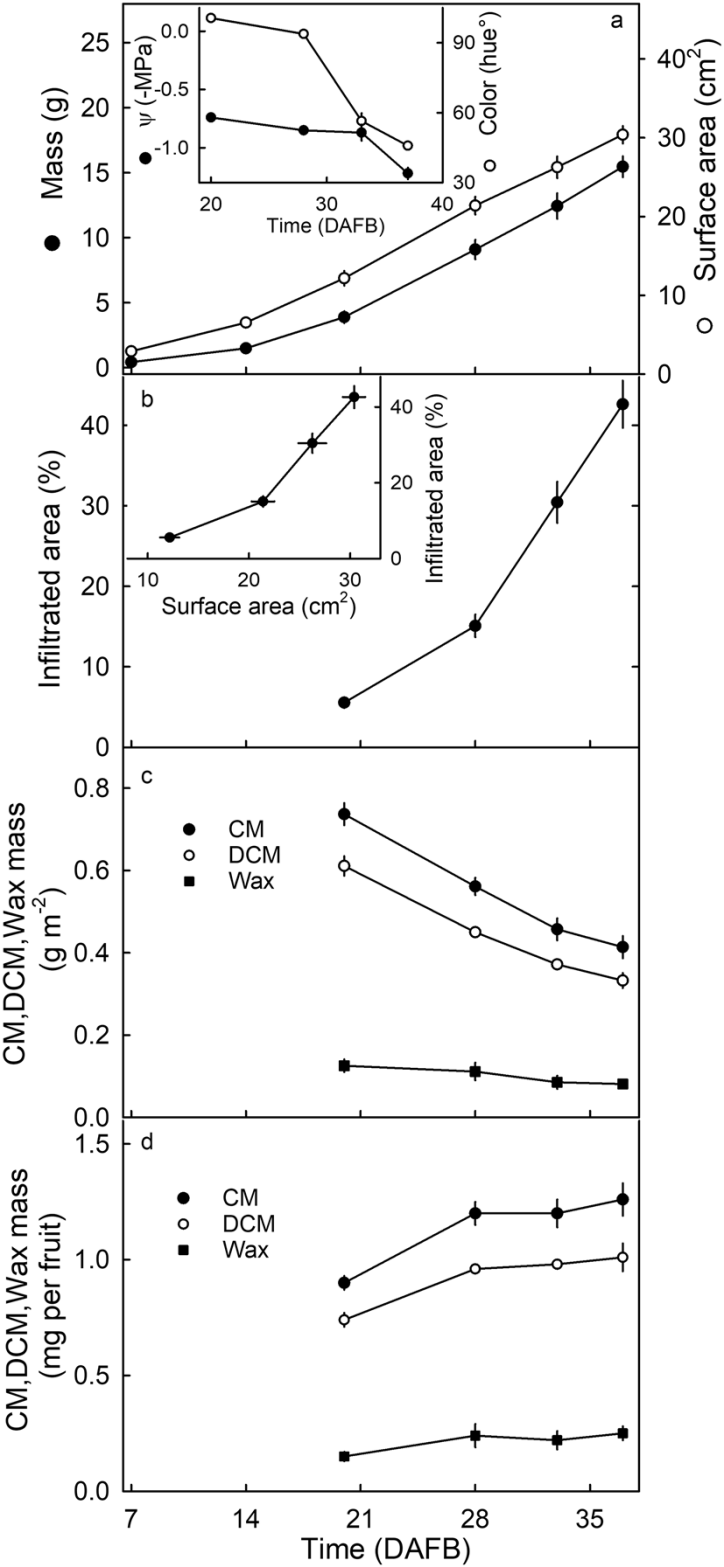
Developmental time course of change in (**a**) fruit mass, (**b**) microcracking of the cuticular membrane, (**c**) mass per unit area of the CM, the dewaxed CM (DCM), and the wax, and (**d**) mass per fruit of the CM, DCM and wax of ‘Clery’ strawberry. Inset in a: Developmental time course of change in color and osmotic potential (Ψ). Inset in b: Microcracking as a function of fruit surface area. Microcracking was indexed by the area infiltrated with acridine orange. The mass per fruit of the CM, DCM and wax was calculated by multiplying the mass per unit area by the surface area of a whole fruit.

During strawberry fruit development, fruit mass and fruit surface area increased sigmoidally with time (Fig. 4a, main graph). At the same time, the receptacle color changed from green (≈100° Hue), to white (≈94° Hue) and to red (≈45° Hue) as hue angle decreased (Fig. 4a, inset). The change in color from green to white began before fruit ripening, the subsequent change to red occurred during ripening.

Cuticular microcracking as indexed by the percentage of the surface area infiltrated by the fluorescent tracer acridine orange, increased during development (Fig. 4b, main graph). The relationship between the percentage infiltrated area and fruit surface area was biphasic. When small fruit grew, there was little increase in microcracking. However, beyond about 28 DAFB microcracking increased markedly as fruit surface area increased (Fig. 4b, inset).

Change in the mass per unit surface area of the isolated CM served as a proxy for change in cuticle thickness. On this basis, cuticle thickness decreased during development (Fig. 4c). The decrease in unit CM mass was due primarily to a decrease the unit DCM mass. This says that unit cutin matrix mass decreased more than unit wax mass. On a whole-fruit basis, total CM mass increased by 33% from flowering (0 DAFB) to the white stage (28 DAFB) and then remained substantially constant until ripeness (Fig. 4d).

**Figure 5:**
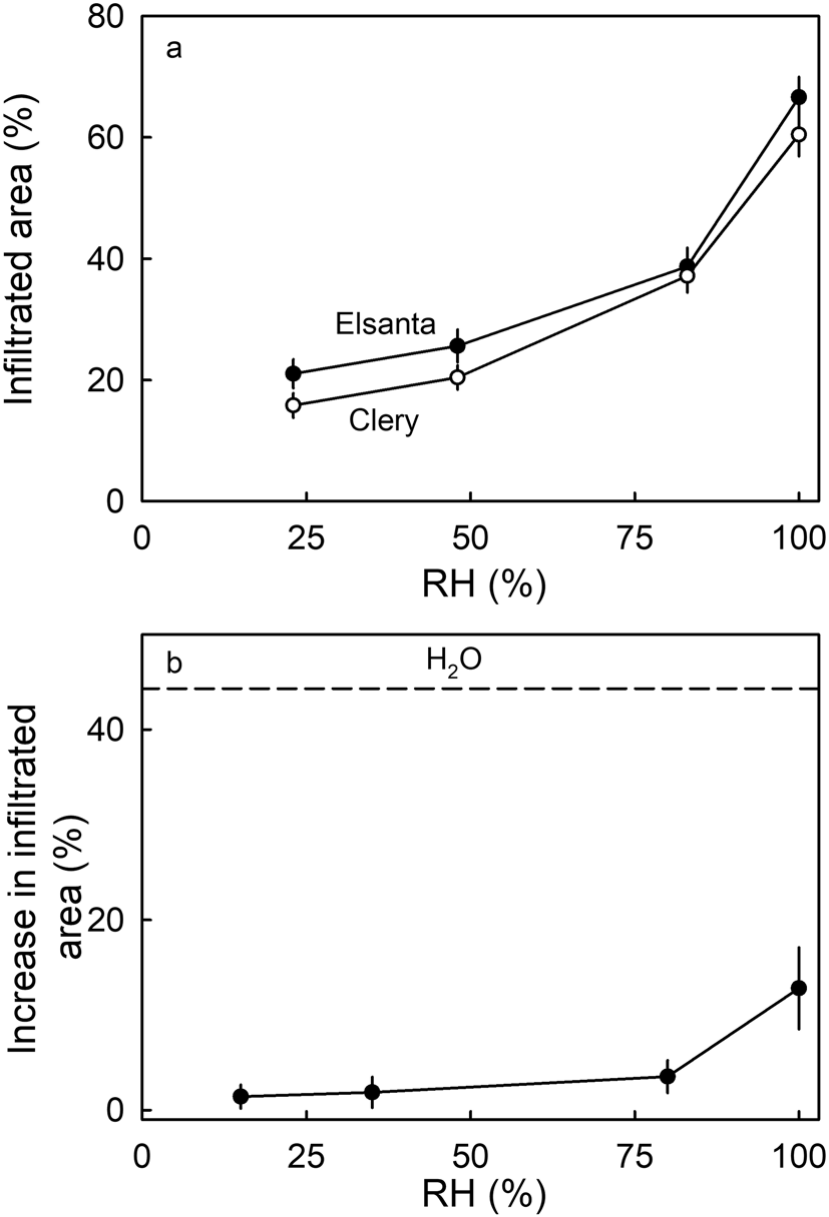
(**a**) Effect of relative humidity during fruit development on microcracking of the cuticle of ‘Elsanta’ and ‘Clery’ strawberries. Fruit were incubated in pottles for RH control between 10 days after full bloom and full ripeness. The bottom of the pottles was covered with dry silica gel, CaCl_2_, NaCl or deionized water. Using this setup, the RH in the pottle equilibrated at about 23% RH when the bottom of the pottle was covered with dry silica gel, at 48% RH when using CaCl_2_, at 83% RH when using NaCl and at 100% RH when using deionized water. At the ripe stage the percentage of the fruit surface area infiltrated with acridine orange was quantified. For experimental setup see supplementary Fig. S4. (**b**) Effect of relative humidity during a 24 h incubation period on microcracking of the cuticle of ripe ‘Clery’ strawberry. Microcracking was indexed by the increase in area infiltrated with acridine orange before and 24 h after exposure to water. For experimental setup see supplementary Fig. S4.

The severity of cuticular microcracking on ripe fruit depended on the RH environment of the fruit during development (Fig. 5a). When fruit were grown at higher RHs, cuticular microcracking increased exponentially (Fig. 5a). This effect was common between the two strawberry cultivars investigated. As RH increased, so too did microcracking, particularly for RH values above 75% (Fig. 5b).

**Figure 6:**
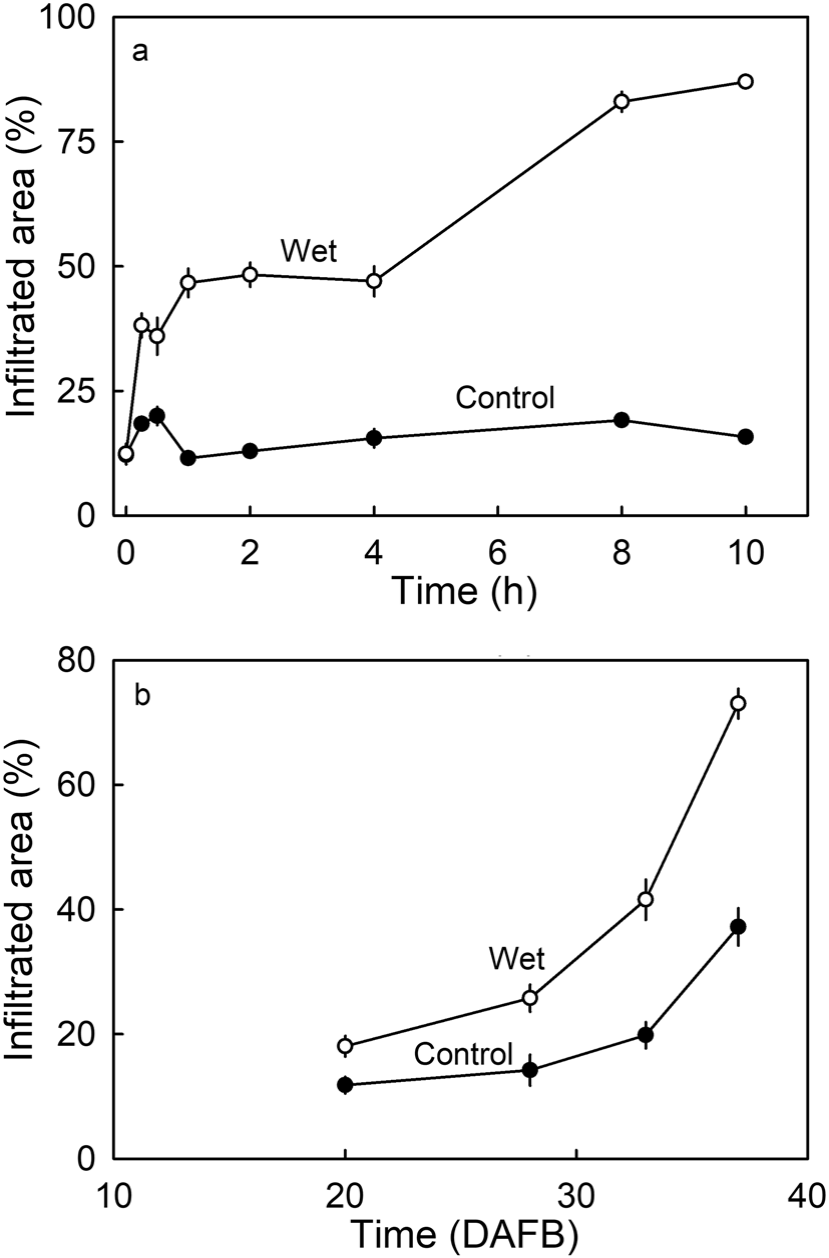
(**a**) Time course of moisture-induced microcracking of the cuticle of ‘Elsanta’ strawberry. Half of the fruit (along the longitudinal axis) was incubated in deionized water (‘Wet’), the other half remained in the air (‘Dry’) and served as control (‘Control’). (**b**) Developmental time course of moisture-induced microcracking of the cuticle of ‘Elsanta’ strawberry as indexed by the area infiltrated with acridine orange. The fruit was incubated for 4 h in deionized water as described for (**a**).

Vapor-phase water (RH) was less effective in increasing microcracking than liquid-phase water. Exposure of the fruit surface to liquid water, increased cuticular microcracking rapidly, compared to the opposing fruit surface (control) that remained dry (Fig. 6a). Surface exposure to liquid water also induced microcracking in younger fruit (Fig. 6b). As with the cuticle’s response to water vapor exposure (Fig. 5a), susceptibility to liquid water exposure increased towards maturity (Fig. 6b).

## Discussion

The main findings of this study were: (1) Growth strain is the primary driver of microcracking of strawberry’s very-thin cuticle and (2) exposure of the fruit surface to water—either vapor or liquid—greatly exacerbates cuticular microcracking.

### Growth strain is the primary driver for cuticular microcracking

Growth strain causes cuticular microcracking. First, cuticle synthesis and deposition cease at about 28 DAFB, when the fruit has achieved about half its later maximum surface area (at ripeness). The subsequent doubling of fruit surface area to the fully-ripe stage, has the effect of distributing an approximately constant amount of cuticle over about twice the surface area. As a result, the cuticle is significantly strained and this strain is associated with significant thinning and failure. Interestingly, the cessation of cuticle deposition (28 DAFB) coincides with a breakpoint in microcracking incidence and severity. Before this stage, the deposition of cutin and the impregnation of the cutin network with wax partially ‘fixed’ the accumulating strain^6,16^. The fixation of strain (elastic) essentially prevented/reduced microcracking. Beyond the breakpoint, the microcracking increased markedly, probably as a result of the continuing increases in strain in the face of the absences of any deposition of new cutin and wax and, hence, the absence of any further strain ‘fixation’. Unfortunately, the thin, and extremely fragile strawberry cuticle, prevented a strain-relaxation analysis^17,18^. However, it is expected that the increase in strain and the concurrent decrease in structural support by the underlying tissue due to fruit softening, induced failure and microcracking of the CM^8^. This cause and effect relationship is not unique to strawberry but is also seen in grapes^19^, sweet cherries^20^ and plums^21^.

The above interpretation is also consistent with the following arguments. First, the strawberry cuticle is the thinnest fruit cuticle ever reported across large number of fruit species^14^. This makes it very susceptible to failure. Second, microcracks tend to be more frequent in the fruit’s proximal region (calyx) and equatorial region, than in its distal region (tip) (*P* = 0.06). The proximal and equatorial regions have larger diameters than the narrower tip region, due to the higher rates of radial expansion of the fruit. For similar reasons one would predict larger fruit will show more microcracking than smaller fruit. However, this relationship was not evident in our results—perhaps because of high fruit:fruit variability in microcracking. Third, the unique surface topography of the strawberry causes a characteristic pattern of microcracking. To better understand the pattern of microcracking, it is important to understand the origins of the unusual ‘corrugated’ topography of the strawberry surface. The corrugation of the surface results from restricted flesh expansion growth in the immediate vicinity of an achene and the lack of such a restriction in the spaces between achenes. The restriction in flesh expansion is probably a result of tensile forces in the radially orientated vascular bundles serving the achenes, with the result that the achenes are ‘pulled’ into the surface as flesh expansion growth proceeds. This results in the ‘funnel type’ structure of the achene cavity where the vascular bundles form the center of the funnel. This interpretation is consistent with a pronounced parallel radial orientation of the epidermal cells (in depressions and on the rim) irrespective of the position of the achene on the fruit surface. As a consequence, microcracks form transverse to this extension growth as the cuticle is dragged along by the underlying epidermal cells. This explains the peaks in the orientation frequency at ± 90° relative to any radius drawn from the achene axis.

However, the above explanation does not account for the about 20% incidence of microcracks in the achene depression orientated parallel to the radii (0°) and the about 30% incidence on the rims between the depressions. Interestingly, in the achene depression these parallel-orientated microcracks occurred in both the proximal (calyx) and the equator regions but not in the distal (tip) region. A parallel orientation of the microcracks would result from a ‘flattening’ of the funnel-shape of the achene depression and thus an increase in the depression diameter. This would be consistent with a tangential strain as fruit volume and, hence, fruit circumference increased^22^.

Finally, there was preferential location of microcracks above the periclinal epidermal cell walls (compared with above the anticlinal cell walls) in the achene depressions and on the rims between depressions. This distribution indicates strain is greater above the periclinal walls. This result is similar to that in sweet cherry, where microcracking is not related to an underlying anticlinal cell wall^8^. This is different from apple, where the pattern of microcracking aligns with the underlying anticlinal epidermal cell walls, indicating that a failure of cell–cell adhesion is a factor in microcracking^23^.

### Both liquid- and vapor-phase water trigger microcracking

Surface moisture plays a critical role in cuticular microcracking in strawberry fruit. Microcracking was especially increased when the fruit surface was exposed to liquid-phase water and less so, but still significantly, when it was exposed to high concentrations of vapor-phase water. These findings are not unique to strawberry but have also been reported in apple^24^, mango^7^, grape^9^ and sweet cherry^25^.

The mechanism of water-induced microcracking is not known. Earlier experiments demonstrated that strawberry fruit incubated in an isotonic polyethylene glycol solution did not take up water but they did develop microcracks^12^. Hence, water uptake by the epidermal cells is judged unlikely to play a direct role. What other factors might be involved in water-induced microcracking?

First, exposure to liquid water, or even to high concentrations of vapor-phase water, hydrates the cuticle^26^. It is already known that hydration of a strained cuticle alters its rheological properties, leading to a general weakening. Both the cuticle’s stiffness and its fracture force decrease, while its fracture strain increases. This phenomenon has been demonstrated for the cuticles of sweet cherry^25^, tomato^27^ and for apple and pear^28^.

Second, it is possible that surface wetness (liquid-phase water) may induce swelling of the epidermal cell walls immediately underlying the cuticle. Cell wall swelling would strain the immediately overlying area of cuticle and so could result in microcracking. Epidermal cell wall swelling has been reported in strawberry during ripening^29^. Here, the swelling is accompanied by an increase in intercellular spaces, presumably due to a reduction in cell–cell adhesion and to partial cell separation in the strained fruit skin^29^. In sweet cherry, cell wall swelling results in decreased cell–cell adhesion^30^. Additional evidence for a role for cell wall swelling in microcracking in strawberry comes from an effect of Ca ions on microcracking. Microcracking is reduced if fruit are incubated in a CaCl_2_ solution^31^. In sweet cherry, Ca ions decrease cell wall swelling by increasing crosslinking of the cell wall constituents. This particularly applies to the homogalacturonans of the middle lamella^32^. As a consequence, cell–cell adhesion is increased and along with the skin’s fracture force^30,33^. These interpretations are consistent with the absence of a role for the cuticle in fruit-skin mechanics. It is the epidermal and hypodermal cell layers that represent main structural member in most fruit skins—not the cuticle^34–36^.

Lastly, surface wetness (liquid-phase water) decreases the rate of cuticle deposition (both wax and cutin) and the decrease favors cuticle failure during fruit expansion growth. In apple, the genes involved in cuticle synthesis and deposition are downregulated when the fruit surface is exposed to surface moisture^37^. It is not known if such downregulation also occurs in strawberries.

## Conclusion

The strawberry cuticle is subject to marked growth strain during fruit development. First, this is because the rate of cuticle deposition in strawberry is low, compared with other fruit crop species, and it is also limited to the early stages of fruit development. Second, strawberries develop to considerable size during quite a short period of time. As a result, the amount of cuticle present at the stage deposition ceases must be stretched over a much larger area during quite a short period of time. In the light of this information, the high incidence of cuticular microcracking in strawberry is hardly surprising. Taking both a whole-fruit perspective, and also a more local achene-depression perspective, the incidences and orientations of microcracking are fully consistent with the idea that growth strain is the dominant driver of cuticular microcracking. Along with this, as in many other fruit crop species, the exposure of the strained cuticle of strawberry to either liquid-phase or vapor-phase water exacerbates microcracking. Projecting these mechanistic understandings to the production issues facing strawberry growers, the cultivation of strawberries in protected environments or in regions with low rainfall and low RH and the maintenance of open canopy structures should all reduce cuticular microcracking and hence also reduce the incidences of water soaking, flesh cracking and fruit rots.

## Material and methods

### Plant material

Strawberry (*Fragaria* × *ananassa* Duch., ‘Clery’, ‘Elsanta’ and ‘Florentina’) were cultivated in a growth chamber or in a greenhouse on the Herrenhausen Campus of the Leibniz University, Hannover, Germany (lat. 52° 23′ N, long. 9° 42′ E). Plants in the growth chamber were grown in pots (14 cm diam.) filled with a peat/moss-based growing medium (Einheitserde Pikiererde Typ P; Company, Sinntal-Altengronau, Germany). The growing conditions in the growth chamber were set at 20/16 °C day/night temperature, 60/80% day/night relative humidity (RH), and a 16 h photoperiod. In the greenhouse, plants were grown in boxes (size 25 × 120 × 20 cm) with 8–10 plants per box. Fruits were selected for uniformity of size and color and absence of macroscopically visible defects.

### Microscopy

Microcracking of the cuticle of strawberry fruit was studied following infiltration with the fluorescent tracer acridine orange using fluorescence microscopy. When incubating fruit in an aqueous solution of acridine orange, penetration of the tracer is restricted to openings in the cuticle including those associated with microcracks, lenticels, and (sometimes) stomata and trichomes^8^. When viewed at the appropriate wavelength, tissues infiltrated with acridine orange exhibit orange, yellow and green fluorescence^8^. Orange, yellow and green fluorescence indicates decreasing concentrations of fluorescent tracer. Fruit was incubated in 0.1% (w/w) aqueous acridine orange (Carl Roth, Karlsruhe, Germany) for 5 min. This incubation time is sufficiently long to allow dye uptake, but not long enough to induce microcracks. After incubation, fruit was rinsed with deionized water, blotted dry with soft tissue paper, and the fruit surface viewed under a fluorescence binocular microscope (MZ10F; Leica Microsystems, Wetzlar, Germany). Calibrated images were taken randomly (Camera DP71; GFP-plus filter, 480–440 nm excitation, ≥ 510 nm emission wavelength) in the equatorial region of the fruit, where diameter is at its maximum. The number of individual fruit replicates was 20. Microcracking was indexed by recording the percentage of the surface area in the microscope window infiltrated by acridine orange. The areas infiltrated by acridine orange were quantified using image analysis (cellSens Dimension 1.18; Olympus Soft Imaging Solutions, Münster, Germany). Fluorescing areas associated with achenes and trichomes were subtracted.

## Experiments

### Characterizing microcracking

*Orientations of microcracks and epidermal cells in the achene depressions and on the rims between depressions.* The achene depression, and the rim between adjacent depressions, were selected because they are representative locations on the ‘corrugated’ strawberry fruit surface. The orientations of microcracks and epidermal cells were quantified as the angle made between the microcrack, or of the long axis of an epidermal cell, and a radius drawn from that point to the point of attachment of the achene—like the spokes of a bicycle wheel, with the point of attachment of the achene being the hub (cellSens Dimension 1.18; Olympus Soft Imaging Solutions, Münster, Germany). Depending on the orientation of a microcrack or cell, the angle value might range from − 90° (anticlockwise) to + 90° (clockwise). The exact point of attachment of an achene was determined in a preliminary experiment in which achenes were excised and viewed under a microscope with the lower side up so that the point of attachment was visible (VHX-7000; Keyence, Osaka, Japan). Calibrated images were taken and the maximum length (y_max_) and maximum width (x_max_) of the achene determined using image analysis. The point of attachment was then expressed relative to the y_max_ and the x_max_. This point had the x and y coordinates of 0.5·x_max_ and 0.7·y_max_ (Supplementary Fig. S3). This point was then used as the hub for the radius measuring the microcrack or epidermal cell orientation.

The frequency distributions of the angles of orientation of microcracks and epidermal cells in the achene depressions and on the rim between depressions were established in three regions along the fruit’s longitudinal axis: in the calyx region (proximal); the equatorial region (maximum fruit diameter and about the center of the fruit), and the tip region (distal).

The dimensions of the achene depressions (length, width, depth) were measured on 3D-images using a digital microscope (VHX-7000; Keyence, Osaka, Japan). The areas of the fruit surface investigated were the proximal, equatorial, and distal regions of the fruit. The fruit used for these measurements were selected to be of almost the same mass (10.9 ± 0.2 g). The number of replicates was 16 per region.

#### Effect of fruit size on microcracking

This was studied by harvesting 20 fruits of the same ripeness stage (color) but of a broad range of different masses (5.1–26.4 g per fruit). The soluble solids of the expressed juices were quantified (mean 8.4 ± 0.4° Brix; CV = 20.0%) using a refractometer (DR6200-T; A. Kruess Optronic, Hamburg, Germany). To record microcracks, the procedure described above was used.

#### Microcracking in different regions along the fruit

Microcracking was also quantified in three different regions of the fruit surface along its length: within the region carrying achenes near the calyx (proximal end), near the equator (the point of maximum diameter) and near the tip (distal end). A total of 14 fruits were investigated.

#### Microcracking above the anticlinal and periclinal epidermal cell walls

To identify the sites of initiation of microcracking, it was assumed the shortest microcracks had only recently been initiated. We selected only those shorter than the dimensions of the underlying epidermal cells. The percentages of short microcracks lying just above the anticlinal cell walls and those just above the periclinal cell walls were recorded in the achene depressions and also on the rim between adjacent depressions.

### Factors affecting microcracking

#### Microcracking and development

The time course of cuticular microcracking was investigated in fruit sampled at 20, 28, 33 and 37 days after full bloom of the respective flower (DAFB). Younger fruit (< 20 DAFB) were too small to measure. Microcracks were quantified following infiltration with acridine orange as described above. Fruit developmental stage was characterized by measuring fruit mass, color and the osmotic potential of the expressed juice. Color was quantified in the equatorial region using a spectrometer (CM-2600 d, orifice 3 mm diameter; Konica Minolta, Tokyo, Japan). The hue angle was calculated^38^. Juice of the fruit was extracted using a ‘garlic press’ and its osmotic potential quantified by water vapor pressure osmometry (VAPRO5600; Wescor, Logan, Utah, USA).

The change in mass of the CM, the mass of the dewaxed CM (the DCM) and the wax fraction were determined on the same batches of fruit. Epidermal skin segments (ES) comprising cuticle, epidermis, and adhering flesh were excised in the equatorial region of the fruit using a biopsy punch (4 mm diameter; Kai Europe, Solingen, Germany). Care was taken to include not more than one achene per ES. The CMs were enzymatically isolated by incubating the ESs in a solution of pectinase (90 ml l^−1^; Panzym Super E flüssig, Novozymes A/S, Krogshoejvej, Bagsvaerd, Denmark), and cellulase (5 ml l^−1^; Cellubrix L; Novozymes A/S) buffered in 50 mM citric acid buffer^39^. The pH was adjusted to pH 4.0 using NaOH. Sodium azide (NaN_3_) was added at a final concentration of 30 mM to prevent microbial growth. The isolation medium was refreshed once. Six CM discs per fruit were collected from a total of 25 fruits. There was no significant difference in CM area after isolation when only one ES (7.2 ± 0.2 mm^2^) was excised per fruit or when several ES (7.4 ± 0.2 mm^2^) were excised per fruit. Following isolation, the CMs were rinsed three times with deionized water. Adhering cellular debris was removed by ultrasonication at 35 kHz for 10 min (RK 510; Sonorex Super, Bandelin electronic, Berlin, Germany). Achenes were carefully removed from the CM discs by hand. The CM samples (n = 5 discs per rep) were dried above silica gel for 48 h and weighed on a microbalance (M2P; Sartorius, Göttingen, Germany). Wax was extracted from the CMs by incubating in CHCl_3_/MeOH (1:1, v/v) for 24 h at room temperature. The DCM were dried above silica gel and re-weighed. The wax mass per unit area was obtained by subtracting the mass of the DCM from that of the CM. The wax content was calculated. The number of replicates was 10.

#### Relative humidity and development

The effect of the RH during development on cuticular microcracking was established by containing still-attached and growing fruit in plastic pottles until ripeness (120 ml, PET, screw-cap lids). This was done as follows: at 10 DAFB fruitlets were introduced through a 12-mm-diameter hole in the lid of the pottle. The space between the hole and the peduncle was then sealed with soft plastic-foam plug. This prevented damage of the peduncle and also reduced water–vapor transfer into/out of the pottle^40^. Data logger sensors were placed inside the pottle to monitor humidity (MSR147WD, sensor: FH2.3/160; MSR Electronics, Seuzach, Switzerland). Pottle humidities were adjusted using dry silica gel, or saturated solutions of CaCl_2_, NaCl or deionized water^41^. Using our setup, the RH in the pottle equilibrated at about 23% RH when the bottom of the pottle was covered with dry silica gel, at 48% RH when using CaCl_2_, at 83% RH when using NaCl and at 100% RH when using deionized water. There was no contact between the salts / solutions and the fruit (Supplementary Fig. S4). The salt solutions and silica gel were refreshed twice a week. Ripe fruit were harvested, and microcracks quantified as described above. The number of replicates was 15, where one replicate represented one plant with one pottle per plant.

#### Relative humidity and microcracking

The effect of atmospheric RH on microcracking at maturity was determined using ES^25^. Briefly, stainless steel washers (6.4 mm inner diameter) were glued onto the fruit surface in the equatorial region of a mature strawberry using a fast-curing epoxy glue (UHU Plus Schnellfest; UHU). The washer prevented any strain relaxation of the fruit skin before recording microcracking. The extent of microcracking was quantified before and after exposure to the different humidities. Before exposure, acridine orange was applied to the ES surface. After 5 min, the dye solution was removed, the surface rinsed with deionized water and the ES viewed under a fluorescence microscope. The area infiltrated by dye was quantified. Thereafter, the ES were incubated in polypropylene boxes above either dry silica gel, or saturated salt slurries of CaCl_2_ or NaCl, or deionized water^41^ (Supplementary Fig. S4). Fruit that was submerged in deionized water served as control. After 24 h, the ESs glued to the washers were re-inspected for microcracks, as described above. This procedure allowed quantification of the change occurring in the infiltrated area (microcracking area) of each individual ES during incubation at a particular humidity. The number of replications was 10.

#### Time course of moisture-induced microcracking

A total of 80, visually-similar strawberry fruit were partly submerged in deionized water. They were held in such a way that each fruit’s longitudinal axis was exactly horizontal, and its lower half was wet and its identical upper half was dry (control). After 0, 0.25, 0.50, 1, 2, 4, 8, and 10 h, 10 fruit were examined by staining with acridine orange, blotting dry, and inspecting for microcracks using fluorescence microscopy. The fluorescing areas were quantified as described above.

#### Moisture induced microcracking during fruit development

Moisture effect on microcracking was studied at four stages of fruit development. Fruit was harvested at 20, 28, 33 or 37 DAFB. Individual fruits were incubated for 4 h in deionized water as described above with half being submerged in deionized water and half remaining dry (control). Microcracks were quantified as described above.

### Data analyses

All experiments comply with relevant institutional, national, and international guidelines and legislation. All experiments had completely randomized designs. Data were subjected to analysis of variance and regression analysis using R (version 3.5.1; R Foundation for Statistical Computing, Vienna, Austria). Means were compared using Tukey’s studentized range test at *P* < 0.05. Data in the Tables and Figures are presented as means ± standard errors.

### Supplementary Information

Supplementary Information 1.

Supplementary Figures.

Supplementary Table S1.

## Data Availability

The datasets generated during the current study are available from the corresponding author upon reasonable request.

## References

[1] Domínguez E, Heredia-Guerrero JA, Heredia A (2011). The biophysical design of plant cuticles: An overview. New Phytol..

[2] Jeffree, C. E. Structure and ontogeny of plant cuticles. In *Biology of the Plant Cuticle,* (eds. Riederer, M. & Müller, C.) (Blackwell Publishing, 1996).

[3] Domínguez E, Heredia-Guerrero JA, Heredia A (2017). The plant cuticle: Old challenges, new perspectives. J. Exp. Bot..

[4] Kerstiens G (1996). Cuticular water permeability and its physiological significance. J. Exp. Bot..

[5] Knoche, M. & Winkler, A. Rain-induced cracking of sweet cherries. In *Cherries: Botany, Production and Uses* (eds. Quero-García, J., Lezzoni, A., Puławska, J. & Lang, G.) 140–165 (CAB International, 2017).

[6] Khanal BP, Grimm E, Finger S, Blume A, Knoche M (2013). Intracuticular wax fixes and restricts strain in leaf and fruit cuticles. New Phytol..

[7] Athoo TO, Winkler A, Owino WO, Knoche M (2022). Surface moisture induces microcracks and increases water vapor permeance of fruit skins of mango cv. Apple. Horticulturae.

[8] Peschel S, Knoche M (2005). Characterization of microcracks in the cuticle of developing sweet cherry fruit. J. Am. Soc. Hortic. Sci..

[9] Becker T, Knoche M (2012). Water induces microcracks in the grape berry cuticle. Vitis.

[10] Knoche M, Grimm E, Winkler A, Alkio M, Lorenz J (2019). Characterizing neck shrivel in European plum. J. Am. Soc. Hortic. Sci..

[11] Underhill SJR, Simons DH (1993). Lychee (*Litchi chinensis* Sonn.) pericarp desiccation and the importance of postharvest micro-cracking. Sci. Hortic..

[12] Hurtado G, Knoche M (2021). Water soaking disorder in strawberries: Triggers, factors, and mechanisms. Front. Plant Sci..

[13] Hurtado G, Knoche M (2023). Necked strawberries are especially susceptible to cracking. PeerJ.

[14] Hurtado G, Grimm E, Brüggenwirth M, Knoche M (2021). Strawberry fruit skins are far more permeable to osmotic water uptake than to transpirational water loss. PLoS One.

[15] Jarvis WR (1962). The infection of strawberry and raspberry fruits by *Botrytis cinerea* Fr.. Ann. Appl. Biol..

[16] Si Y, Khanal BP, Schlüter OK, Knoche M (2021). Direct evidence for a radial gradient in age of the apple fruit cuticle. Front. Plant Sci..

[17] Lai X, Khanal BP, Knoche M (2016). Mismatch between cuticle deposition and area expansion in fruit skins allows potentially catastrophic buildup of elastic strain. Planta.

[18] Knoche M, Lang A (2017). Ongoing growth challenges fruit skin integrity. Crit. Rev. Plant Sci.

[19] Becker T, Knoche M (2012). Deposition, strain, and microcracking of the cuticle in developing ‘Riesling’ grape berries. Vitis.

[20] Grimm E, Peschel S, Becker T, Knoche M (2012). Stress and strain in the sweet cherry skin. J. Am. Soc. Hortic. Sci..

[21] Knoche M, Peschel S (2007). Deposition and strain of the cuticle of developing European plum fruit. J. Am. Soc. Hortic. Sci..

[22] Nobel PS (1999). Physicochemical & Environmental Plant Physiology.

[23] Knoche M, Khanal BP, Brüggenwirth M, Thapa S (2018). Patterns of microcracking in apple fruit skin reflect those of the cuticular ridges and of the epidermal cell walls. Planta.

[24] Khanal BP, Imoro Y, Chen YH, Straube J, Knoche M (2021). Surface moisture increases microcracking and water vapour permeance of apple fruit skin. Plant Biol..

[25] Knoche M, Peschel S (2006). Water on the surface aggravates microscopic cracking of the sweet cherry fruit cuticle. J. Am. Soc. Hortic. Sci..

[26] Chamel A, Pineri M, Escoubes M (1991). Quantitative determination of water sorption by plant cuticles. Plant Cell Environ..

[27] Petracek PD, Bukovac MJ (1995). Rheological properties of enzymatically isolated tomato fruit cuticle. Plant Physiol..

[28] Khanal BP, Grimm E, Knoche M (2013). Russeting in apple and pear: A plastic periderm replaces a stiff cuticle. AoB Plants.

[29] Redgwell RJ (1997). In vivo and in vitro swelling of cell walls during fruit ripening. Planta.

[30] Brüggenwirth M, Knoche M (2017). Cell wall swelling, fracture mode, and the mechanical properties of cherry fruit skins are closely related. Planta.

[31] Hurtado G, Knoche M (2022). Calcium ions decrease water-soaking in strawberries. PLoS One.

[32] Schumann C, Winkler A, Knoche M (2022). Calcium decreases cell wall swelling in sweet cherry fruit. Sci. Rep..

[33] Schumann C, Winkler A, Brüggenwirth M, Köpcke K, Knoche M (2019). Crack initiation and propagation in sweet cherry skin: A simple chain reaction causes the crack to ‘run’. PlosOne.

[34] Khanal BP, Knoche M (2017). Mechanical properties of cuticles and their primary determinants. J. Exp. Bot..

[35] Brüggenwirth M, Fricke H, Knoche M (2014). Biaxial tensile tests identify epidermis and hypodermis as the main structural elements of sweet cherry skin. AoB Plants.

[36] Khanal BP, Knoche M (2014). Mechanical properties of apple skin are determined by epidermis and hypodermis. J. Am. Soc. Hortic. Sci..

[37] Straube J (2020). Russeting in apple is initiated after exposure to moisture ends: Molecular and biochemical evidence. Plants.

[38] McGuire RG (1992). Reporting of objective color measurements. HortSci.

[39] Orgell WH (1955). The isolation of plant cuticle with pectic enzymes. Plant Physiol.

[40] Winkler A, Fiedler B, Knoche M (2020). Calcium physiology of sweet cherry fruits. Trees.

[41] Wexler A (1995). Constant Humidity Solutions.

