# Supplementary material for: Microcracking of strawberry fruit cuticles: mechanism and factors
Source: Sci Rep. 2023 Nov 8;13:19376. doi: 10.1038/s41598-023-46366-8 (PMC10632442; doi:10.1038/s41598-023-46366-8)
Supplement: Supplementary file 2 — Supplementary Figures. [file 41598_2023_46366_MOESM2_ESM.docx]

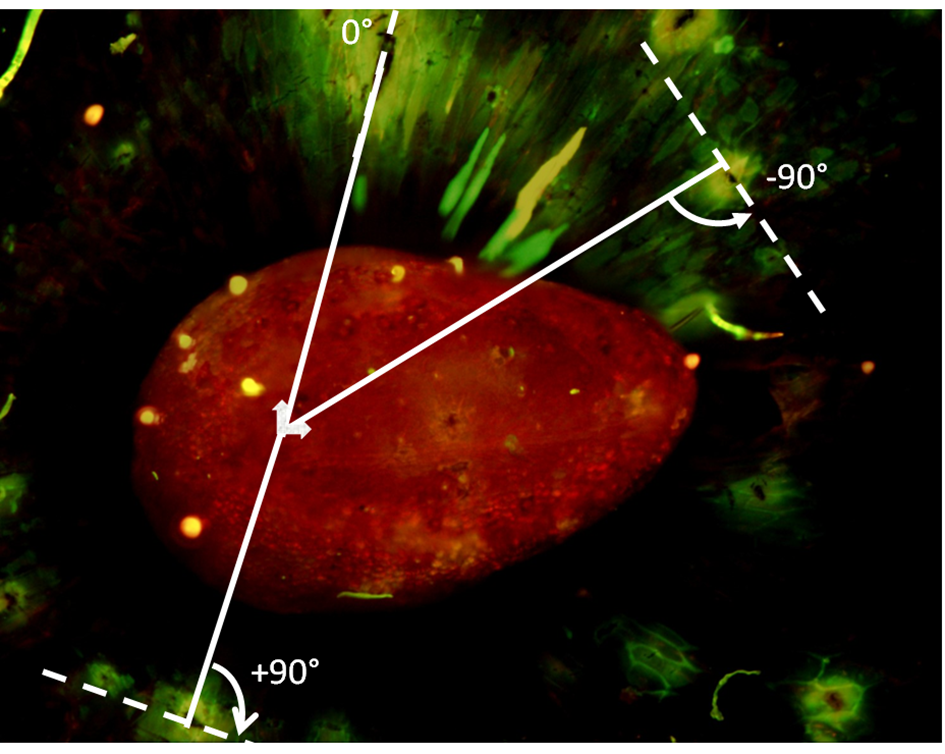


**Supplementary Fig. S1.** Measurement of the orientations of microcracks as the angle made between the microcracks, and a radius drawn from the point of attachment of the achene.





**Supplementary Fig. S2.** (a) Effect of fruit size on microcracking of the cuticle. (b) Microcracking of the cuticle in different regions of ripe ‘Clery’ strawberry. The regions were the calyx-end of the fruit within the seed zone; region of equator (maximum fruit diameter and the center of the fruit), and tip of the fruit. Microcracking was indexed by the area infiltrated with acridine orange.


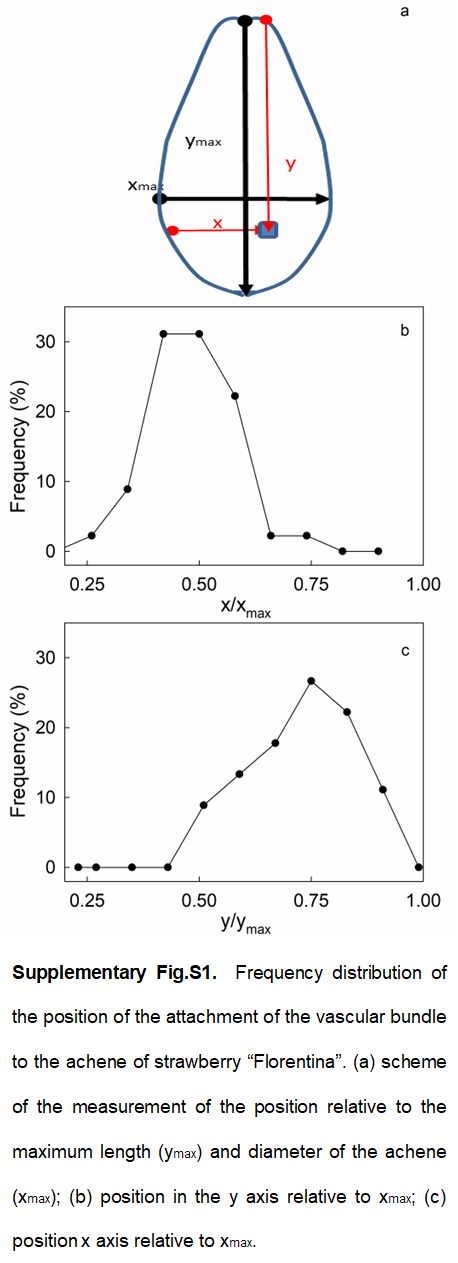


**Supplementary Fig. S3.** Frequency distribution of the position of the attachment of the vascular bundle to the achene of strawberry ‘Florentina’. (a) Scheme of the measurement of the position relative to the maximum length (Y_max_) and diameter of the achene (X_max_). (b) position on the x axis relative to X_max_. (c) position on the y axis relative to Y_max_.


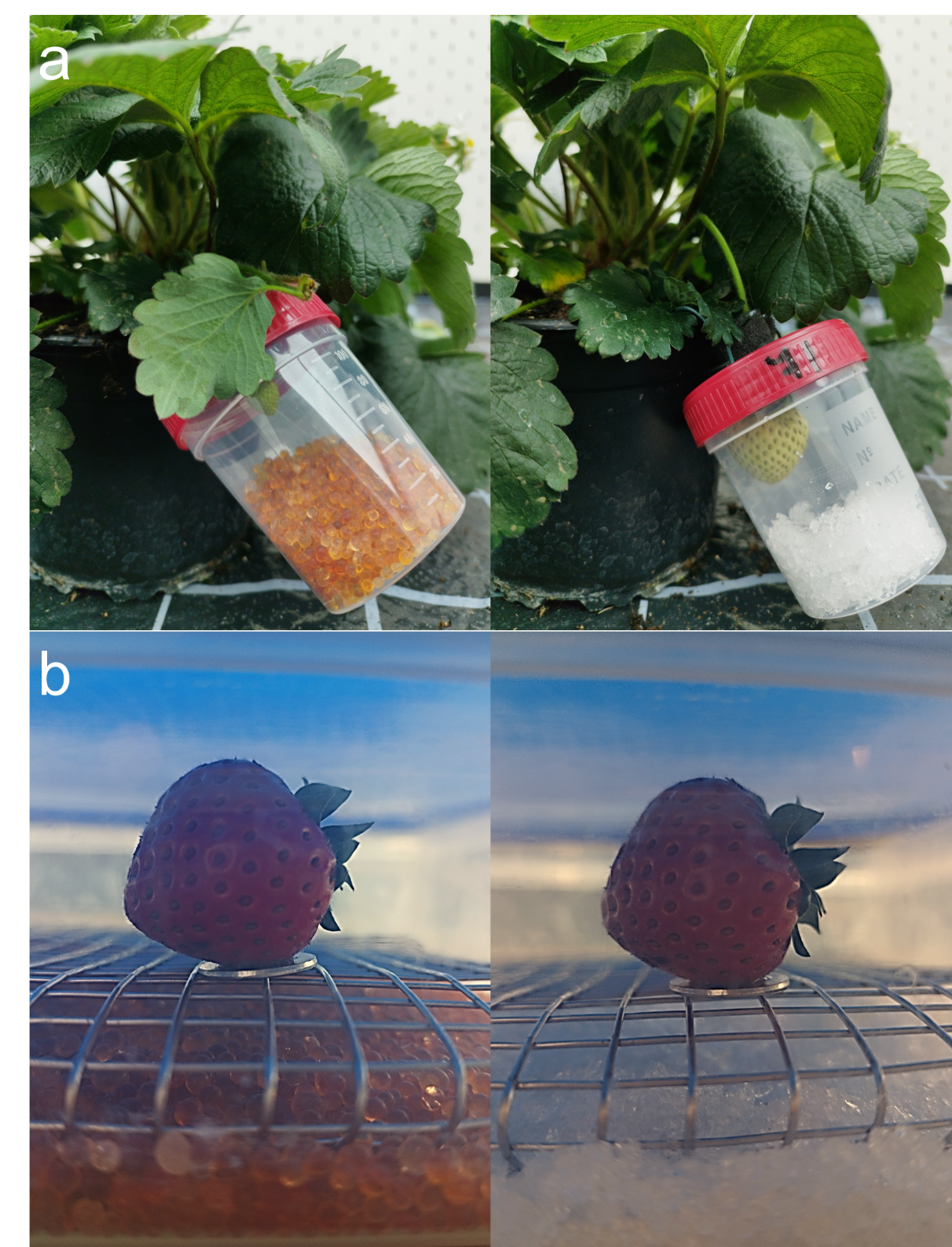


**Supplementary Fig. S4.** (a) Experimental setup of the study on the effect of the relative humidity (RH) during fruit development on cuticular microcracking; (b) Experimental setup of the study on the effect of RH on microcracking at maturity.
