# Supplementary material for: Microcracking of strawberry fruit cuticles: mechanism and factors
Source: Sci Rep. 2023 Nov 8;13:19376. doi: 10.1038/s41598-023-46366-8 (PMC10632442; doi:10.1038/s41598-023-46366-8)
Supplement: Supplementary file 3 — Supplementary Table S1. [file 41598_2023_46366_MOESM3_ESM.docx]

|  | Calyx-end | Equator | Tip |
| --- | --- | --- | --- |
| Depth (um) | 727±39 | 806±35 | 798±27 |
| Length (um) | 2945±101 | 3077±110 | 3091±90 |
| Width (um) | 2531±99 | 2712±109 | 2625±84 |
| Volume (mm^3^) | 2.3±0.3 | 2.8±0.3 | 2.6±0.2 |
| Ratio (W/L) | 0.9±0.0 | 0.9±0.0 | 0.9±0.0 |
| **Supplementary Table S1.** Dimensions of the cavity depression within the calyx-end (proximal), equator (equatorial), and tip (distal) regions of strawberry fruit ‘Clery’. | | | |
